# American Veterinary College—Commencement

**Published:** 1892-04

**Authors:** 


### AMERICAN VETERINARY COLLEGE—COMMENCEMENT.

The 17th Annual Commencement services of the American Veterinary College were held at Chickering Hall, New York, on Thursday evening, March 24th, 1892. The hall was crowded to standing room, with an interested audience of more than ordinary attractive appearance, which included a large percentage of pretty ones in petticoats, who were in harmony with the floral decorations and the music by Cappa's 7th Regiment Band, and who showed how popular the embryo veterinarians are.

Fanuil D. Weisse, M. D., President of the Board of Trustees, conferred the Degree of D. V. S. upon the following gentlemen:

Harry Webster Acheson, Washington, D. C.; James Edgar Assing, New York, N. Y.; Ulysses S. Grant Bieber, Kutztown, Pa.; Wendel Valentine Bieser, New York, N. Y.; Charles Richard Borden, Taunton, Mass.; Thomas Earle Budd, Woodbury, N. J.; John Campbell, New York, N. Y.; Carlisie Norwood Drake, Guttenberg, N. J.; Robert Dickson, New York, N. Y.; William Henry Dodge, Leominster, Mass.; Bruce Linwood Drummond, Woodbridge, N. J.; Charles Frank Dwinal, Mechanic Falls, Me.; Andrew Young Earl, Honeoye Falls, N. Y.; John Thomas Ferley, Jr., Philadelphia, Pa.; Charles Hugo Ford, Moorefield, W. Va.; Jacob Homer Gardner, New York, N. Y.; James Alfred Haas, Lyon Valley, Pa.; Arthur Julius Hammerstein, St. Louis, Mo.; Horace Albert Hedrick, Cockeysville, Md.; Michael F. Hoar, Springfield, Mass.; Joseph Rhodes Hodgson, Jr., Brooklyn, N. Y.; James Henry Honan, Delphi, Ind.; George Washington Hood, New York, N. Y.; John Bogert Hopper, Ridgewood, N. J.; Abel Sackville Richard Jones, New York, N. Y.; Theodore Aloysius Keller, New York, N. Y.; William Bennett Kelley, Holtsville, L. I.; Bernard Paul Kenny, New York, N. Y.; William Arminus Koke, Brooklyn, N. Y.; John Eugene Kramer, San Antonio, Tex.; Louis Henry Kraus, New Haven, Conn.; Horace Deland Lambert, Salem, Mass.; John Stilwell Lamkin, Loveland, Col.; Frank Kirk Nice, Germantown, Pa.; William Arthur Nickel, Brooklyn, N. Y.; Frederick Adrien Nief, Paris, France; William Lawrence Nunan, Coriz, Pa.; Adam Wilson Ormiston, Germantown, Pa.; José Maria Peralta, Cartago, C. A.; Charles Wyman Shaw, Kingston, Mass.; Thomas Whaley Shaw, Worcester, Mass.; Albert James Sheldon, Naugatuck, Conn.; Edward Jacob Shipsey, New York, N. Y.; De Witt Clinton Smith, Greensburg, Ind.; John David Sturm, Dana, Ind.; William Rogers Tatum, Glenelg, Md.; William Franklin Woolston, Victor, N. Y.

An admirable valedictory by Albert James Sheldon, D. V. S. of the graduating class, showed how thoroughly he and his classmates appreciated the gravity of the life upon which they were about to enter. The Rev. Roderick Terry, D. D., followed with an address.

The officers of ths class were: Pres., John B. Hopper; Vice-Pres., Albert J. Sheldon; Sec'y, Edward J. Shipsey; Treas., Ulysses G. Bieber; Class Historian, Carlisle N. Darke; Marshall, T. Earle Budd. The Committee of Arrangements were : James S. Lamkin, Joseph R. Hodgson, Jr., Charles R. Borden, Charles H. Ford, Thomas W. Shaw, Albert J. Burkholder, Robert Dickson, Charles F. Dwinal, J. Edward Rowe, Jr., Louis H. Kraus, José M. Peralta, Harry W. Acheson, James E. Assing. Messrs. Joseph B Quin, Chief; Ernest L. Volgenau, John W. Mackey, Fred. L. Ray, Frank J. Williamson, William W. Yard, Jr., William C. Ferguson, Charles Martin, James L. Lindsay, Harry F. Steele, Joseph H. Hutchinson, Richard F. Dwyer, and Geo. C. Bretherton, of the Junior class, served as ushers.

Later in the evening a banquet was given at Clark's by the Alumni Association; at which a majority of the graduating class and a large number of visitors were present. Toasts were responded to by Drs. Robertson, Liautard, Huidekoper, Hoskins and others.

